# Apple Vision Pro and Its Implications in Mohs Micrographic Surgery: A Narrative Review

**DOI:** 10.7759/cureus.71440

**Published:** 2024-10-14

**Authors:** Alan D Kaye, Rahib K Islam, Kazi N Islam, Amor Khachemoune, Christopher Haas, Sonnah Barrie, Alberto Pasqualucci, Sahar Shekoohi, Giustino Varrassi

**Affiliations:** 1 Anesthesiology, Louisiana State University Health Sciences Center, Shreveport, USA; 2 Medicine, Louisiana State University School of Medicine, New Orleans, USA; 3 Agricultural Research Development Program, Central State University, Wilberforce, USA; 4 Dermatology, State University of New York Downstate Medical Center, Brooklyn, USA; 5 Dermatology, Louisiana State University Health Sciences Center New Orleans, New Orleans, USA; 6 Anesthesia and Critical Care, University of Perugia, Perugia, ITA; 7 Pain Medicine, Fondazione Paolo Procacci, Rome, ITA

**Keywords:** apple vision pro, augmented reality, dermatological surgery, intraoperative guidance, mixed reality, mohs micrographic surgery, postoperative analysis, preoperative planning, surgical precision, technological advancements

## Abstract

Mohs micrographic surgery (MMS) is a precise and effective technique for treating skin cancers, necessitating high accuracy and real-time decision-making to ensure complete tumor removal while preserving healthy tissue. The introduction of the Apple Vision Pro (AVP), an advanced mixed-reality headset, presents a potential technological advancement in surgical practice. The present investigation explores the integration of AVP into Mohs surgery, focusing on its potential to enhance preoperative planning, intraoperative guidance, and postoperative analysis. The AVP’s high-resolution displays, advanced eye-tracking technology, and real-time data overlay capabilities may assist in meticulous surgical planning and execution. In the preoperative phase, AVP enables the creation and manipulation of detailed 3D models, providing comprehensive visualizations of tumor margins and critical structures. During surgery, AVP’s real-time data overlay aids in precise tumor excision and spatial orientation, reducing errors and improving outcomes. Postoperatively, the AVP facilitates detailed procedural reviews and serves as a robust educational tool for surgical trainees. Despite its promising applications, challenges such as real-time resolution limitations, potential eye strain from prolonged use, and high costs must be addressed for widespread clinical adoption. This review highlights the transformative potential of AVP in Mohs surgery and underscores the need for further research to optimize integration and maximize benefits in dermatological procedures.

## Introduction and background

Mohs micrographic surgery (MMS) is a precise surgical technique employed to treat skin cancer. Developed by Dr. Frederic E. Mohs in the 1930s, this procedure involves systematic removal and microscopic examination of cancerous tissues, layer by layer, until only cancer-free tissue remains [1]. The meticulous nature of MMS requires high levels of accuracy and real-time decision-making to ensure complete cancer removal while preserving as much healthy tissue as possible [2].

MMS is regarded as the benchmark for removing cancerous skin tissue and is commonly used to treat prevalent skin cancers such as squamous cell carcinoma and basal cell carcinoma [3]. The introduction of advanced surgical technologies has consistently expanded possibilities for precision and improved patient outcomes. Among these innovations, extended reality (XR) technologies, including virtual reality (VR, a simulated, immersive digital environment that allows users to engage in an artificial world), augmented reality (AR, allows for stacking of virtual information and objects on to the real world, enhancing the user's point of view and interaction with their environment), and mixed reality (MR, a technology that blends the physical and digital worlds, allowing real and virtual elements to interact and coexist in real-time, creating a hybrid environment), have demonstrated considerable potential in improving various surgical procedures. One example of this advanced technology is the Apple Vision Pro (AVP), a state-of-the-art mixed-reality headset that seamlessly blends digital information with the physical environment. Launched by Apple Inc. in early 2024, the AVP boasts features like high-resolution displays, advanced eye-tracking, and real-time data overlay as listed on their website, positioning it as a transformative tool in various medical fields, including surgery [4,5].

Recent applications of AVP in fields such as plastic surgery, ophthalmology, and neurosurgery have demonstrated its potential to enhance surgical precision, improve educational outcomes, and streamline intraoperative procedures [6]. In plastic surgery, for instance, the AVP has been used for detailed anatomical simulations and enhanced surgical planning, significantly improving surgeon accuracy and patient outcomes [7]. Similarly, in ophthalmology, XR has facilitated advanced diagnostic and therapeutic procedures, improved patient care, and expanded the possibilities for vision restoration [8,9]. Neurosurgery has also benefited from AVP’s capabilities, with surgeons using the technology for preoperative planning and real-time anatomical visualization, thereby enhancing the precision and safety of complex procedures [10,11]. Given these advancements, exploring the potential of AVP in MMS is both timely and crucial. This review aims to assess how AVP can be integrated into MMS to enhance preoperative planning, intraoperative guidance, and postoperative analysis. By leveraging the capabilities of XR, Mohs surgeons can achieve higher precision in tumor excision, greatly improve and increase healthy outcomes for patients, and foster a more efficient and educational surgical environment. The present investigation, therefore, explores the benefits, challenges, and future directions of incorporating AVP into MMS, highlighting its potential to revolutionize dermatological procedures. Any feature listed on the APV is found under technical specifications on Apple’s website.

## Review

Technological overview of Apple Vision Pro

The AVP represents a significant leap forward in the integration of XR into medical and surgical applications. Its technological capabilities are designed to offer unparalleled precision, efficiency, and immersive experiences that can significantly enhance the practice of MMS.

Features and specifications

One of the standout features of the AVP is advanced eye-tracking technology. This system uses multiple infrared cameras to accurately track the user’s gaze, allowing for precise interaction with virtual elements overlaid on the real world. This capability is crucial in a surgical setting where even slight deviations in focus can impact the accuracy of the procedure [12].

The AVP runs on VisionOS, a dedicated operating system specifically optimized for mixed-reality applications in clinical and surgical environments. VisionOS enhances performance through its ultra-low latency capabilities, which are critical for real-time responsiveness during surgical procedures. Low latency ensures that virtual overlays, such as 3D models or surgical guides, remain perfectly aligned with the surgeon’s perspective, reducing any risk of misalignment that could occur with delayed feedback.

Moreover, VisionOS incorporates precise eye-tracking algorithms, leveraging Apple’s proprietary infrared technology, which allows for accurate gaze-based control. This precise control is vital for tasks such as manipulating 3D models or accessing patient information hands-free, thus maintaining a sterile environment. The operating system is powered by Apple’s M2 and R1 chips, which provide the computational power necessary for high-performance processing, enabling smooth and efficient operation even during extended and complex surgical applications [7,9].

By supporting a wide range of applications from iOS to iPadOS, VisionOS provides a robust and versatile platform for developers to create specialized medical applications tailored for surgical use. This flexibility allows for the integration of cutting-edge software solutions that can be directly applied to enhance surgical precision and efficiency [4,7].

Mixed reality capabilities

The MR capabilities of the AVP are another critical aspect that makes it suitable for use in MMS. The device features high-resolution 4K displays with a passthrough latency of under 12 milliseconds, enabling real-time overlay of digital information onto the physical surgical field. This low latency is essential for maintaining the surgeon’s spatial awareness and ensuring that virtual enhancements do not lag behind real-world actions, which is vital for accuracy during delicate procedures like tissue removal [7].

The AVP’s ability to overlay digital images and data in real time significantly enhances preoperative planning and intraoperative guidance compared to traditional methods. Traditional approaches often rely on static 2D imaging, which provides limited perspective and requires the surgeon to mentally reconstruct the 3D spatial relationships of anatomical structures. In contrast, the AVP allows for the integration of dynamic 3D models directly into the surgeon's field of view, offering a comprehensive and interactive visualization of patient anatomy. This 3D overlay can be rotated, zoomed in/out, and aligned with the surgical site, enabling a more intuitive and detailed understanding of the surgical area [7,10].

For example, in MMS, where precise mapping of cancerous tissues and clear margins is critical, the AVP’s real-time 3D visualization ensures that the surgeon has an accurate and immediate representation of the surgical site. This reduces the cognitive load associated with interpreting traditional 2D imaging and allows for precise, data-driven adjustments during surgery, ultimately improving surgical accuracy and patient outcomes [10].

Real-time data overlay and manipulation

The AVP also excels in providing real-time data overlay and manipulation, which can significantly enhance the surgical workflow. Surgeons can access patient data, imaging results, and other critical information without having to look away from the surgical site. This seamless integration of digital data with the physical environment helps maintain focus and reduces the likelihood of errors caused by shifting attention [12].

Furthermore, the AVP’s sophisticated hardware, including its array of cameras and sensors, supports advanced spatial computing. This allows the device to accurately map the physical environment and track the surgeon’s movements, enabling precise interaction with virtual objects [11]. For example, during MMS, the AVP can be used to project probable tumor margins based on preoperative imaging, assisting the surgeon in making more informed decisions during tissue excision. The AVP’s advanced features, including eye-tracking, high-resolution displays, low-latency passthrough, and real-time data overlay, make it not only an innovative but powerful tool for enhancing MMS by equipping surgeons with the precision and efficiency needed for more effective procedures.

Applications in MMS

The integration of the AVP into MMS holds promise for significant advancements in preoperative planning, intraoperative guidance, and postoperative analysis. The AVP's capabilities can enhance precision, efficiency, and educational outcomes in this highly detailed surgical procedure.

Preoperative planning

One of the most critical phases of MMS is preoperative planning, where detailed visualization of the patient's anatomy is essential. The AVP can be utilized to create and manipulate 3D models of the patient’s skin and underlying tissues, allowing surgeons to plan their approach with unparalleled accuracy. These 3D renderings provide a comprehensive view of the tumor margins, surrounding healthy tissue, and any critical structures that need to be preserved [13].

Comparison to Traditional Methods

In traditional preoperative planning, surgeons use forms of 2D imaging that may include MRI or CT scans to get a sense of not only tumor size but its location as well [14]. However, these methods are limited by their inability to provide effective, interactive, real-time 3D visualizations [15]. The AVP, in contrast, offers dynamic 3D modeling that surgeons can utilize from a multitude of different dimensions and perspectives, such as having the ability to rotate, zoom, and explore from different angles. This offers a deeper understanding of tumor depth and its relationship with nearby anatomical structures, leading to anatomical fluency [16]. This level of interaction helps surgeons avoid intraoperative surprises. It reduces the need for guesswork, which is particularly important when operating in areas with complex anatomy, such as around the eyes, nose, or ears [17].

Integration With Imaging Technology

The AVP can seamlessly integrate with imaging data from MRI, CT, and other diagnostic tools, allowing for more detailed planning. For example, as touched on before, by overlaying the 3D tumor model on the physical anatomy in real-time, surgeons can visualize tumor depth and surrounding vasculature, nerve pathways, and other critical structures. This could be vital for a Mohs surgeon operating on cosmetically sensitive areas such as the face, which has many sensitive areas [18]. This enhanced visualization minimizes the risk of damaging vital structures and reduces the likelihood of complications, such as excessive tissue removal or the need for follow-up surgeries [19].

Personalized Surgery Simulation

Another advantage is the AVP’s ability to offer patient-specific simulations. Surgeons can practice the procedure in a virtual environment before the surgery, experimenting with different approaches to determine the safest and most effective course of action. This can provide synergy with any current forms of simulation exercises already in place for these surgeons [20]. In cases of recurring or aggressive skin cancers, this preoperative rehearsal can be crucial for achieving clean margins while preserving cosmetic outcomes [21]. Personalized simulations enable surgeons to pre-plan incisions and tissue removal with a precision that was previously impossible, tailoring the procedure to each patient’s unique anatomy.

Enhanced Collaboration and Multitasking

The AVP’s multitasking capability also enhances and even encourages collaboration. Surgeons can consult with colleagues in real time while manipulating 3D models, ensuring that the preoperative strategy is optimized from multiple perspectives. For example, a Mohs surgeon might consult a reconstructive plastic surgeon during planning to ensure that tumor excision and subsequent reconstruction are addressed seamlessly. In addition, even during the procedure, the AVP can contact attending or chief surgeons if any questions arise through voice control. The AVP also supports simultaneous viewing of multiple applications, such as patient history, imaging data, and surgical guidelines, ensuring that surgeons have all the necessary information without disrupting the planning flow [12]. There are many cases that have tried this existing feature, with many benefits [22].

Future Potential for AI Integration in Preoperative Planning

In the future, AI could be integrated with the AVP to augment preoperative planning further. Machine learning algorithms could analyze tumor characteristics and predict the most likely locations for margin involvement, suggesting optimal excision paths for surgeons [23]. AI could also provide real-time feedback based on previous surgeries, recommending adjustments to improve precision based on known outcomes for similar cases [24]. Combining AVP’s immersive visualizations with AI-driven recommendations could take surgical planning to the next level. Interacting with these models in a virtual environment enables surgeons to simulate different scenarios and optimize their surgical strategy before the actual procedure begins. The view of multiple apps at once offered by the AVP further proves useful for this, as multitasking is made much more efficient and can be helpful for surgical strategy regarding the procedure. The AVP's technical specifications were received from the official Apple website.

Intraoperative guidance

The real-time capabilities of the AVP are particularly beneficial during the intraoperative phase of MMS. The device can overlay digital information directly onto the surgeon's field of view, providing continuous guidance throughout the procedure. For example, AVP can display tumor margins based on preoperative imaging and real-time tissue mapping, ensuring that surgeons excise all cancerous tissues while preserving as much healthy tissue as possible [12,25]. This level of precision is crucial in MMS, where the goal is to achieve clear margins with minimal tissue removal. In addition to this, notes can be made to stay open during the procedure, from notes about the patient to any notes made regarding the procedure, or even from textbooks. Furthermore, as the AVP operates on VisionOS, a form of iOS, one can even communicate very efficiently and effectively with other surgeons during the procedure. Being able to quickly access this information via the unique operating software that it is on is what sets this apart from other headsets and devices.

Additionally, the AVP can enhance the surgeon's spatial orientation by providing a detailed, augmented view of the surgical site. This feature helps in maintaining a clear understanding of the anatomical landmarks and the extent of tissue removal required, thereby reducing the likelihood of errors and improving overall surgical outcomes. The AVP's real-time data overlay can also include vital information such as patient vitals, imaging results, and procedural checklists, which can be accessed without diverting attention from the surgical field [26].

Postoperative analysis

Postoperative analysis and educational review are integral components of continuous improvement in surgical practice. The AVP can be used to record and review the entire surgical procedure in 3D, providing a valuable resource for postoperative analysis. Surgeons can revisit the recorded procedure to assess their technique, identify areas for improvement, and ensure that all cancerous tissues are effectively removed [27].

This capability is also beneficial for training and education. Surgical trainees can use the AVP to virtually step into the operating room and observe complex procedures from the surgeon's perspective [28]. The immersive experience provided by the AVP enhances learning and retention, making it an effective tool for surgical education. Trainees can also practice their skills in a simulated environment, gaining confidence and proficiency before performing actual surgeries [12].

Educational tool for postoperative review and training

The AVP’s application extends beyond the operating room into the realm of education and training. By leveraging its immersive capabilities, the AVP can be used to create detailed educational modules that simulate real-life surgical scenarios. Trainees can interact with these modules, practicing their skills in a controlled, risk-free environment [29]. Additionally, the AVP can facilitate remote education, allowing experts to guide and mentor trainees from different locations, thus expanding access to high-quality surgical training. The AVP has the potential to revolutionize how surgeons are trained. Surgical trainees can use AVP to explore patient anatomy virtually, practicing various incision techniques and tumor excision strategies that may be difficult and require practice. This virtual training environment reduces the learning curve for complex procedures like MMS, making it a valuable educational tool. Furthermore, residents could simulate different tumor presentations and tissue depths, giving them practical experience that mirrors real-life scenarios [30].

Discussion

Technological Limitations

While the AVP offers numerous advantages, it is not without its technological limitations. One significant challenge is the current real-time resolution and accuracy of the data overlay during surgical procedures. Although the AVP provides high-resolution displays and advanced eye-tracking technology, the precision required for highly detailed and delicate procedures such as MMS may still be compromised under certain conditions. For instance, slight calibration issues or latency fluctuations, even if minimal, can impact the alignment of virtual overlays with the physical anatomy, potentially affecting surgical accuracy. This may necessitate further refinement of the system to ensure that real-time updates remain consistently accurate throughout procedures [5].

Additionally, prolonged use of the AVP could lead to issues such as eye strain or fatigue. Given the intensity and duration of surgical procedures, surgeons may experience visual discomfort or headaches, which are critical considerations when using AR systems. Prolonged focus on MR displays, especially when overlaid with intricate digital information, may strain the eye muscles, impacting the surgeon's performance and concentration. To mitigate these effects, improvements in display comfort, refresh rates, and adaptive lighting could be beneficial.

The physical weight of the headset, currently around 600 grams, also poses a challenge for extended use. While the device is designed to be ergonomic, wearing it for several hours may cause neck strain or discomfort, which could affect the surgeon’s mobility and precision during lengthy operations. Addressing this limitation would involve reducing the device's weight or providing additional support mechanisms to minimize physical fatigue over time [9,12].

Integration Into Clinical Practice

Integrating the AVP into clinical practice involves several logistical and financial considerations. The initial cost of the AVP, which can be up to $5000 with accessories, may be prohibitive for some clinics and healthcare providers [12]. This financial barrier could limit the widespread adoption of technology, particularly in smaller or underfunded medical facilities.

Adapting the AVP for specific clinical environments and workflows is another challenge. Surgical teams will need to undergo training to effectively utilize the new technology, which requires time and resources. Additionally, the AVP must be seamlessly integrated into existing surgical protocols and practices to ensure it enhances rather than disrupts the surgical workflow [28].

Ethical and Regulatory Issues

The implementation of AVP in surgical practice raises important ethical and regulatory issues. Ensuring patient confidentiality and data privacy is paramount, as the AVP involves capturing and processing sensitive patient information [31]. It is important to ensure that adequately effective security measures are in place to protect this data from access that is not authorized [7].

Informed consent is another critical consideration. Patients must be fully informed about the use of XR technology in their surgical procedures and any potential associated risks or benefits [32]. Establishing clear guidelines and regulatory frameworks is essential to address these ethical concerns and safeguard patient rights in the age of digital medicine [12].

Technological Improvements

Future technological advancements will pave the way to overcoming the current limitations of the AVP. Enhancements in real-time resolution and interaction capabilities are essential to fully realizing the potential of XR in surgical applications. Improving the comfort and ergonomics of the headset will also be vital for long-term use during surgeries. Developing specialized surgical applications and software explicitly tailored for MMS and other dermatological procedures will further enhance the utility of the AVP [33]. Collaboration between medical professionals and technology developers will be key to creating solutions that meet the specific needs of surgical practice [9].

Broader Clinical Integration

As technology matures, expanding the use of AVP to other dermatological surgeries and medical specialties can provide broader benefits. For instance, its application in reconstructive and aesthetic surgeries has already shown promise, and similar benefits can be extended to various other surgical fields [7,9,10]. Potential collaborations between tech developers and medical professionals can accelerate the pace of accurate surgical software development. Engaging with engineers and developers to create customized solutions will ensure that the technology evolves in ways that are most beneficial to clinical practice [5].

Apple Vision Pro’s Distinction in a World of Mixed Reality Devices

The AVP differs from traditional XR devices through means of how it displays information, as it is based on cameras instead of regular displays. This opens up much higher visualization capabilities due to the extremely high-resolution power of the AVP [11]. As a result, tumor margins can be defined much more precisely during operations. Furthermore, the AVP sets itself apart from other traditional XR devices through means of the “Apple ecosystem” that it is a part of. Due to being a part of iOS, the operating software, this allows for seamless transitions between all the users’ devices, allowing for easy access to patient notes, past surgeries, or any other useful tool to the surgeon, just to name a few examples received from Apple’s official website.

Having notes for the surgery while also being connected to iCloud during the procedure itself can prove to be very useful for the Mohs surgeon. This allows much convenience, which other XR devices do not offer. Furthermore, apps are continually being developed and updated on the app store, which will also make way for more innovation in the future regarding procedures. As a result, the AVP shows its use and distinction in a world of updated technology.

Long-Term Impact Studies

To fully understand the impact of AVP on surgical outcomes and patient care, long-term impact studies and clinical trials are necessary. These studies will help assess the efficacy and safety of the technology in real-world surgical settings. Ongoing evaluation and research will be critical to refining the technology and ensuring it delivers the intended benefits. Additionally, evaluating the impact of AVP on training and education in Mohs surgery will provide insights into its effectiveness as an educational tool. As more data becomes available, it will be possible to optimize the use of AVP in surgical training programs and enhance the learning experience for surgical trainees [28].

Insurance and Accessibility Considerations

Given the potential of the AVP to significantly enhance surgical precision and patient outcomes in MMS, support from insurance companies for the acquisition of such technology and related software development is crucial. Incorporating AVP into medical practice may come with high initial costs, but these could be offset by long-term benefits such as improved surgical accuracy, reduced procedure times, and lower complication rates, which ultimately lead to cost savings for healthcare systems. Insurance coverage for this equipment would not only make it more accessible to healthcare providers but also encourage the integration of innovative technologies that enhance patient care. This investment aligns with the broader goal of improving medical outcomes through advanced technological solutions.

## Conclusions

In conclusion, while the integration of AVP in Mohs surgery brings challenges related to technological refinement, financial investment, and ethical considerations, there is much potential to leave an impact on dermatological surgery. By focusing on critical areas such as technological improvements, the development of clinical guidelines, and long-term impact studies, the future of XR in dermatology, specifically Mohs surgery, looks exceptionally promising. As AVP technology continues to evolve and become more accessible, it is poised to play an integral role in shaping the future of precision-based dermatological care.
